# Vesicoamniotic Shunting Improves Outcomes in a Subset of Prune Belly Syndrome Patients at a Single Tertiary Center

**DOI:** 10.3389/fped.2018.00180

**Published:** 2018-07-03

**Authors:** Jeffrey T. White, Kunj R. Sheth, Aylin N. Bilgutay, David R. Roth, Paul F. Austin, Edmond T. Gonzales Jr., Nicolette K. Janzen, Duong D. Tu, Angela G. Mittal, Chester J. Koh, Sheila L. Ryan, Carolina Jorgez, Abhishek Seth

**Affiliations:** ^1^Scott Department of Urology, Baylor College of Medicine, Houston, TX, United States; ^2^Division of Urology, Department of Surgery, Texas Children's Hospital, Houston, TX, United States; ^3^Department of Urology, Emory University School of Medicine, Atlanta, GA, United States; ^4^Memorial Hermann Health System, Houston, TX, United States

**Keywords:** prune belly syndrome, triad syndrome, Eagle-Barrett syndrome, prenatal intervention, mortality, renal failure, pulmonary hypoplasia, orchiopexy

## Abstract

**Objective:** Review outcomes of Prune Belly Syndrome (PBS) with the hypothesis that contemporary management improves mortality.

**Methods:** A retrospective chart review of inpatient and outpatient PBS patients referred between 2000 and 2018 was conducted to assess outcomes at our institution. Data collected included age at diagnosis, concomitant medical conditions, imaging, operative management, length of follow-up, and renal function.

**Results:** Forty-five PBS patients presented during these 18 years. Prenatal diagnoses were made in 17 (39%); 65% of these patients underwent prenatal intervention. The remaining patients were diagnosed in the infant period (20, 44%) or after 1 year of age (8, 18%). Twelve patients died from cardiopulmonary complications in the neonatal period; the neonatal mortality rate was 27%. The mean follow-up among patients surviving the neonatal period was 84 months. Forty-two patients had at least one renal ultrasound (RUS); of the 30 patients with NICU RUSs, 26 (89%) had hydronephrosis and/or ureterectasis. Of the 39 patients who underwent voiding cystourethrogram (VCUG), 28 (62%) demonstrated VUR. Fifty-nine percent had respiratory distress. Nine patients (20%) were oxygen-dependent by completion of follow up. Thirty-eight patients (84%) had other congenital malformations including genitourinary (GU) 67%, gastrointestinal (GI) 52%, and cardiac 48%. Sixteen patients (36%) had chronic kidney disease (CKD) of at least stage 3; three patients (7%) had received renal transplants. Eighty-four percent of patients had at least one surgery (mean 3.4, range 0–6). The most common was orchiopexy (71%). The next most common surgeries were vesicostomy (39%), ureteral reimplants (32%), abdominoplasty (29%), nephrectomy (25%), and appendicovesicostomy (21%). After stratifying patients according to Woodard classification, a trend for 12% improvement in mortality after VAS was noted in the Woodard Classification 1 cohort.

**Conclusions:** PBS patients frequently have multiple congenital anomalies. Pulmonary complications are prevalent in the neonate while CKD (36%) is prevalent during late childhood. The risk of CKD increased significantly with the presence of other congenital anomalies in our cohort. Mortality in childhood is most common in infancy and may be as low as 27%. Contemporary management of PBS, including prenatal interventions, reduced the neonatal mortality rate in a subset of our cohort.

## Introduction

PBS is a rare congenital disorder with a reported incidence of 3.8 cases per 100,000 live male births in the United States (1–3). The classic clinical triad of PBS consists of deficient abdominal wall musculature, urinary tract dilation, and bilateral cryptorchidism (4). PBS has also been referred to as Eagle-Barrett Syndrome (5) or Triad Syndrome (6). While several theories of PBS ontogeny exist, the true mechanism of its development remains unknown (7, 8). Most cases are sporadic, but several familial cases of PBS have been reported, supporting a possible sex-linked autosomal recessive mode of inheritance (9). This disorder is ~18–20 times more common in males than females (10). A female variant with similar abdominal wall and urinary tract findings but without cryptorchidism has been described, which only constitutes ~3% of all PBS cases (7).

The GU phenotype of PBS is variable and does not necessarily correlate with the degree of abdominal wall flaccidity. Most patients develop renal dysfunction due to congenital renal dysplasia and/or scarring from urinary tract infections resulting from ureteric obstruction, VUR, or bladder dysfunction. Forty-to-fifty percent of patients require renal replacement therapy and ~15% require renal transplantation (11). Extra-GU manifestations may be seen as well, including GI, orthopedic, and cardiopulmonary diagnoses (12).

Chronic childhood conditions such as PBS are an economic stress on a family and healthcare system (13). No reports have quantified this economic burden. The tests and procedures required to provide a reasonable quality of life can be daunting, especially with a recent report suggesting that magnetic resonance urography may improve the clinical monitoring of PBS patient population (14). Most importantly, human capital calculations, i.e., wages lost and expenses incurred due to time away from work, have not been reported as in ureteropelvic junction obstruction (15). The development of a standardized clinical guideline for management of PBS patient population would provide an opportunity to plan for such an emotional and financial burden. Such guidelines would empower families and hopefully reduce the rates of early fetal termination for PBS which can be as high as 31% (16, 17).

Because of the rarity of PBS, few studies have reported outcomes; surgical management of PBS remains controversial. Due to long-distance referrals and older patient age, Lopes et al. prefer a comprehensive surgical treatment including concurrent abdominoplasty, upper and lower urinary tract reconstruction, orchiopexy, and circumcision where applicable (18). Alternatively, Seidel et al. prefer a common-sense approach where surgical procedures are dictated by patient problems (11). This report by Seidel et al. is the only contemporary report on management and outcomes of an U.S.-based PBS patient cohort since 1999 (19).

Establishing treatment guidelines for PBS has been challenging due to both the few number of cases and the variable penetrance of the syndrome (12, 18). We present the outcomes and management in a contemporary PBS cohort at a single tertiary center over the past 18 years. We hypothesized that contemporary management, including prenatal intervention, would reduce mortality rates in a single center PBS patient cohort.

## Materials and methods

After obtaining institutional review board approval, we conducted a retrospective chart review of PBS patients presenting to our institution between 2000 and 2018. Forty-five PBS cases were identified by ICD-9/-10 code search (756.71 and Q79.4, respectively) followed by chart review confirmation.

Prenatal referrals with bilateral hydroureteronephrosis and oligo-/anhydramnios were diagnosed with lower urinary tract obstruction (LUTO). These patients were evaluated by our fetal center and stratified by LUTO severity according to Ruano et al. which uses prenatal ultrasound and fetal urine chemistries for staging (31). If indicated, patients underwent vesicoamniotic shunting (VAS).

Patients surviving the perinatal period (fetal period and up to 28 days of age) underwent clinical and functional evaluation on presentation, including ultrasonography and renography (20). Voiding cystourethrogram (VCUG) was performed to assess urethral and bladder pathology including urethral atresia, megalourethra, VUR, and bladder characteristics. Urodynamic evaluations were performed for patients who were unable to void and/or had episodes of urinary retention. Renal function was evaluated by serum urea and creatinine levels.

For each patient, age at diagnosis, comorbid medical conditions, imaging results, operative management, length of follow up, and renal function at last follow up were recorded. Patients were then categorized according to Woodard: Classification I includes patients with bilateral renal dysplasia, oligo-/anhydramnios LUTO, and/or pulmonary hyposplasia; Classification II consists of patients with the typical triad and initially stable renal function that may decline due to recurrent infections and obstruction from the syndromic uropathy; Classification III includes those with triad variants and stable renal function despite the uropathy.

Ethnicity, prematurity (defined as birth at gestational age < 37 weeks), evidence of renal dysplasia on ultrasound, history of recurrent febrile urinary tract infections (defined as 2 or more culture-proven urinary tract infections in 1 year), presence of VUR, and presence of other congenital anomalies were evaluated as potential predictors of mortality and/or development of renal failure (CKD stage 4–5). Univariate statistical analysis was performed using the Chi-square and Fisher exact tests as appropriate to determine potential risk factors for mortality and renal failure with *P* < 0.05 representing statistical significance.

## Results

Forty-five confirmed cases of PBS were included (Table 1). Most of our cohort was male (87%); six patients (13%) were female. Half of our cohort was African American, 20% Caucasian, and 29% Hispanic.

**Table 1:** Patient characteristics.

**Sex**	**N (%)**
M	39 (87)
F	6 (13)
**Ethnicity**	
African American	22 (50)
Caucasian	9 (20)
Hispanic	12 (29)
Unknown	1 (1)
**Prenatal diagnosis**	17 (39)
Prenatal intervention	11 (65)
Mean age at last f/u, in months (range)	84 (2.4-193.0)
**Urological diagnoses**	
VUR	28 (62)
Febrile UTI	17 (39)
Recurrent febrile UTI	5 (11)
Recurrent nonfebrile UTI	4 (15)
Hydronephrosis and/or hydroureter	26 (89)
PUV	11 (25)
Duplication anomaly	2 (7)
Hypospadias	2 (0.05)
CKD ≥ stage 3	16 (36)
**Other congenital malformations**	
Any	40 (91)
Cardiac	21 (48)
Multiple	5 (19)
ASD	4 (9)
Left ventricular non-compaction	1 (0.2)
GI	23 (52)
Imperforate anus	7 (16)
Anal atresia	1 (4)
Malrotation	3 (0.7)
Gastroschisis	1 (4)
CHAOS	3 (0.7)
**Pulmonary insufficiency**	26 (59)
Oxygen Dependent	9 (20)
Deceased, from pulmonary causes	12 (27)

*ASD, atrial septal defect; CHAOS, congenital high airway obstruction. PUV, posterior urethral valves; UTI, urinary tract infection; VUR, vesicoureteral reflux*.

Diagnosis of suspected LUTO was made prenatally in 17 (39%); 65% of these patients underwent prenatal intervention, i.e., VAS (Table 1). Of the 11 patients undergoing VAS, only one required renal transplantation during follow-up. No cases were terminated prenatally. Diagnosis was made in the immediate neonatal period in 20 (45%) and at a later age due to late childhood referrals in 8 (18%). Twelve patients died from cardiopulmonary complications with an average lifespan of 80 days (0–300 days). The neonatal mortality rate in our cohort was 27% (*n* = 12).

Among surviving patients, the mean follow-up was 84.2 months (Table 1). Forty-two patients had at least one RUS, which showed that 26 (89%) of these patients had some degree of hydroureteronephrosis. Thirty-nine patients (87%) underwent VCUG, with 28 (62%) demonstrating VUR.

Most patients (91%) had comorbid congenital anomalies. The most common comorbid anomalies included pulmonary in 59% and GI in 52%. Sixteen patients (36%) developed CKD of at least stage 3; 12 patients (27%) had CKD stage 4–5. Three patients had received renal transplants as of last follow-up; although transplantation was planned for two additional patients. Sixteen patients (36%) developed respiratory distress, most frequently in the neonatal period (58% prior to 1 month postnatal). Nine patients (20%) remained oxygen-dependent at last follow-up.

Excluding prenatal interventions, 84% of patients had at least one surgery (mean 3.4, range 0–6). The most common surgery was orchiopexy (any approach), performed in 71% of patients. This was followed by vesicostomy in 39%, reimplant in 32%, abdominoplasty in 29%, nephrectomy in 25%, and appendicovesicostomy in 21%. Additional surgeries, such as orchiopexy, are scheduled for the future in several patients, but have not yet been performed.

Our cohort demonstrated a spectrum of phenotypes. To compare and contrast patients more effectively we stratified the cohort according to Woodard's classification system (21, 22). Per the Woodard classification system, 31 patients were stratified into category I, 8 into category II, and 5 into category III (Table 2). All Category I patients were male and had the highest mortality rate (35%). Seventy-nine percent of Category I patients had a peak in their serum creatinine levels >1 mg/dL and 71% had a nadir serum creatinine >0.4 mg/dL. Eighteen percent of Category I patients underwent renal transplantation. Ninety percent of the VAS patients met classification I criteria; Category I patients who underwent VAS had a mortality rate of 20% compared to 32% in Category I patients not undergoing VAS (*p* = 0.08). Category I patients had a significantly higher lifetime rate of surgical interventions averaging four procedures per patient (*p* = 0.03). In contrast, 62% of the category II group was female. This cohort demonstrated similar clinical characteristics: a mortality rate of 38%, 60% of patients with peak serum creatinine >1 mg/dL, 60% with nadir serum creatinine >0.4 mg/dL, and a lifetime surgical rate of 3.5 procedures per patient. Only one of the Category II patients underwent VAS and was associated with mortality at 69 days of age. Category III patients had a statistically significantly lower nadir serum creatinine when compared with Category I patients (*p* < 0.05).

**Table 2:** Woodard Classification of the Cohort.

**Woodard classification**	**I**	**II**	**III**
N	31	8	5
Male (%)	100	38	80
Mortality (%)	35	38	0
Mortality in VAS (%)	20	100	^#^
Mortality in Non-VAS (%)	32	29	^#^
Neonatal peak serum creatinine > 1 mg/dL (%)	79	60	20
Average maximum serum creatinine (Std error, mg/dL)	2.08 (0.44)	2.16 (0.68)	1.00 (0.10)
Average nadir serum creatinine (Std error, mg/dL)^‡^	1.00 (0.29)^*^	0.54 (0.12)	0.28 (0.05)^*^
CKD 3^+^ (%)	27	25	0
**HYDRONEPHROSIS**			
NICU (%)	95	100	67
Last follow-up (%)	95	80	33^+^
**DYSPLASIA**			
NICU (%)	89	75	0
Last follow-up (%)	89	60	67
**SEVERE VUR (4-5)**			
NICU (%)	53	50	33
Last follow-up (%)	43	20	0
**SURGICAL RATES (Avg** ± **Stdev)**^‡^	4.0 (1.7)^*^	3.5 (1.6)	1.75 (0.96)^*^
TUA PUV (%)	29	0	0
Nephrectomy (%)	35	20	0
Abdominoplasty (%)	29	25	0
Orchiopexy(%)	71	38	80
Vesicostomy (%)	35	25	25
Ureteral Reimplant (%)	29	38	35
Renal Transplant (%)	18	0	0
Hypospadias (%)	6	13	0
APV/Mitrofanoff (%)	29	13	0

# *No patients in Classification III underwent vesicoamniotic shunting (VAS)*.

‡ *P-value calculated with ANOVA*.

* *P-value < 0.05*.

+ *1 patient with MCDK and contralateral kidney without hydronephrosis*.

Ethnicity, prematurity, renal dysplasia, febrile UTIs, VUR, and other congenital anomalies were not found to be significantly associated with mortality (Table 3). The presence of additional congenital anomalies was the only variable significantly predictive of increased risk of renal failure (Table 3, *p* < 0.05).

**Table 3:** Potential risk factors for mortality and renal failure: *P-*values.

**Factor**	**Mortality**	**Renal failure**
Ethnicity^†^	0.31	0.11
Prematurity^‡^	1	0.23
Renal dysplasia^‡^	1	0.10
Recurrent febrile UTI^‡^	0.56	0.60
VUR^‡^	1	1
Other congenital anomalies^‡^	0.63	0.040^*^

† *P-value calculated with Chi-square test*.

‡ *P-value calculated with Fisher exact test*.

* *P-value < 0.05*.

## Discussion

Optimal treatment of patients with PBS remains controversial. Outcomes data have been sparse in the literature because of the rarity of the condition, with only limited retrospective data available. Management choices and comparison of single institution outcomes are limited by the varying spectrum of the condition. Some authors have proposed comprehensive reconstruction, with concurrent total urinary tract reconstruction, bilateral orchiopexy, and abdominoplasty (18, 23), while others have endorsed limited operative intervention in most cases (24, 25). Alternative therapies such as corset usage have also been sought (26). It is unclear which approach is superior, as there are no studies comparing outcomes of aggressive surgical reconstruction versus conservative management.

Management of PBS in the setting of prenatal diagnosis is perhaps even more controversial. Termination of pregnancy based on prenatal diagnosis of PBS has been reported as high as 31% (16, 17). Conversely, Leeners et al. reported prenatal VAS in four PBS patients and suggested that such intervention may improve outcomes (27). Differentiating PBS from outlet obstruction prenatally is challenging. Because of this, several VAS and fetal cystoscopy studies have included PBS patients along with posterior urethral valves or urethral atresia, unifying these patient groups under the term “lower urinary tract obstruction” (28–31), even though PBS is not necessarily an obstructive process. PBS has been associated with urethral obstruction or atresia (24, 32, 33). In one series, 18% of PBS patients also had urethral atresia, and all surviving fetuses with both conditions had a vesicocutaneous fistula (32). In another report, 57% of PBS patients who underwent VAS had acceptable long-term renal function (defined as creatinine clearance >70 ml/min) and did not require renal replacement therapy (34). These reports suggest that prenatal shunting may in fact benefit the subset of PBS patients who also suffer from LUTO. However, it is impossible to identify this subset of patients prenatally with noninvasive methods. Thus, prenatal intervention in this patient population remains controversial, with no established guidelines.

Historical cohorts have shown high rates of infant mortality and renal failure in PBS patients. Pillion et al. reported a 29% mortality in the first month of life in their cohort of 14 patients, and a 40% rate of renal failure in those who survived (35). Reinberg et al. reported an even higher mortality of 34.4% in their cohort of 32 patients with PBS (36). In this study, all deaths occurred in the neonatal period. Autopsy was performed in 82% (9/11) of patients who died, with findings of diffuse and severe renal dysplasia in 67% (6/9). They observed renal failure in nearly half of those patients who survived infancy, which they attributed to infection and obstruction more so than dysplasia. The mortality in our cohort (27%) was comparable to other historical cohorts. Interestingly, VAS was associated with a 20% mortality rate in Woodard I classification versus a 32% mortality rate in Woodard Classification I patients without VAS. Though this 12% difference only approached significance, the benefit of saving 3 of 25 babies with Woodard I Classification is clinically relevant. The rate of renal insufficiency appears similar between our (36%) and historical cohorts (35, 36). These data suggest that a larger study reviewing the efficacy of VAS in PBS cohorts is needed. Currently, mortality and renal insufficiency remain unfortunate characteristics of the disorder.

Seidel et al. recently published their outcomes in a cohort of 46 PBS patients referred after the neonatal period (11). These authors reported a 39% rate of CKD, similar to the 36% rate in our cohort. Two of their patients (4.3%) were female, compared to six of our patients (13%). The proportion of females in our patient population was higher than expected, which can be explained by the limitations of small observational studies. The most common site of surgical intervention in Seidel's cohort was testicular, which was also the case in our cohort. In contrast, Seidel et al. noted no mortalities in their cohort. This difference may be explained by our inclusion of prenatal and inpatient referrals.

Lopes et al. also published outcomes regarding comprehensive surgical management of 46 PBS patients from South American referrals (18). Their comprehensive management included simultaneous urinary tract reconstruction, abdominal wall repair, bilateral orchiopexy, and circumcision where applicable. They noted a 26% morbidity rate due to surgical complications. Thirty seven percent of patients required reoperations for obstructed megaureter after ureteroneocystostomy, unilateral VUR, recurrence, four redo abdominoplasties, and three failed orchiopexies. There were no mortalities in their cohort, likely due to referral after the perinatal period. With this aggressive surgical management only 10% proceeded to renal failure. The authors should be commended for their low rate of renal failure. It is important to note that their cohort was significantly different: most of their cohort consisted of Class II patients.

Variation in PBS outcomes reported in the literature is multifactorial. Most contemporary studies, except Lopes et al. describe small cohorts without stratification by Woodard classification. The wide spectrum of PBS severity in these smaller cohorts makes their results difficult to generalize. To overcome these limitations, categorization of disease severity was adopted in our cohort to allow for stratification of outcomes. By classifying our cohort, a trend of reduced mortality in Classification I patients who underwent VAS was noted. These results contradict an anecdotal editorial reported by Woodard (37). This editorial was drafted during earlier experiences in VAS when patient selection algorithms had not yet been developed (31). VAS in PBS patients has been criticized because shunting does not address the cause of uropathy in PBS which Woodard equates to a mesenchymal defect during ureteral and bladder development (37). In our cohort, VAS was performed to correct renal mesenchymal defects such as oligohydramnios, fetal ultrasound changes consistent with dysplasia and/or altered fetal urine chemistry. Therefore, the purpose of the VAS intervention of contemporary series may be different than prior historical series (relief of obstruction).

Several limitations and strengths of this study should be reviewed. This single-center retrospective review with a small population is subjected to the biases inherent with this design. We attempted to minimize biases in the patient cohort by including prenatal referrals, newborn inpatients and postnatal outpatient referrals, thereby addressing the current trends of prenatal interventions as well as neonatal mortality. Additionally, the patients were stratified according to Woodard to more easily extrapolate our results. Surgical interventions were reviewed, however, the role of reconstruction in preserving renal function was not addressed. Finally, the rate of comorbid congenital anomalies was high in our population. This high rate is likely due to not restricting the cohort to outpatient referrals.

Despite these limitations, this study is the most contemporaneous review of PBS which includes prenatal to adolescent patients. We emphasized a common-sense based approach to surgical intervention based on patient needs and demonstrated an improvement in mortality with VAS of Woodard Class I patients. Not surprisingly we discovered that comorbid congenital anomalies are associated with increased risk of renal failure. Larger multi-center studies will be needed to assess the role of reconstruction in preventing renal deterioration.

## Conclusions

Optimal management of PBS remains unclear. Even in modern cohorts, the risk of renal failure remains but VAS in a Woodard Class I patient cohort is associated with a 12% reduction in mortality. Most PBS patients will require multiple surgical interventions, with the most common surgical intervention being orchiopexy. Given the rarity and spectrum of PBS, multi-center studies are needed to build larger cohorts to characterize clinical outcomes, determine prognostic indicators, and develop treatment algorithms that should improve outcomes.

## Ethics statement

This study was a retrospective chart review and exempt from human subjects research protocols. This study was carried out in accordance with the recommendations and approval of Baylor College of Medicine's Institutional Review Board and Texas Children's Hospital Regulatory Review Office.

## Author contributions

AB and AS conceptualized the study with significant clinical input from AM, CJ, CK, DR, DT, EG, NJ, and PA. AB, JW, KS, and SR performed retrospective patient chart review. AB, AS, CJ, KS, and JW interpreted data, performed statistical analyses and drafted manuscript. AS and JW performed multiple revisions of manuscript. All authors revised, read, and approved the final manuscript.

### Conflict of interest statement

The authors declare that the research was conducted in the absence of any commercial or financial relationships that could be construed as a potential conflict of interest.

## Abbreviations

ASD: Atrial Septal Defects
APV: Appendicovesicostomy
CHAOS: Congenital High Airway Obstruction Syndrome
CKD: Chronic Kidney Disease
GI: Gastrointestinal
GU: Genitourinary
LUTO: Lower Urinary Tract Obstruction
PBS: Prune Belly Syndrome
PUV: Posterior Urethral Valve
UTI: Urinary Tract Infection
TUA PUV: Transurethral Ablation of Posterior Urethral Valves
VAS: Vesicoamniotic Shunting
VCUG: Voiding Cystourethrogram.
