# Corrigendum: Vesicoamniotic Shunting Improves Outcomes in a Subset of Prune Belly Syndrome Patients at a Single Tertiary Center

**DOI:** 10.3389/fped.2018.00229

**Published:** 2018-08-14

**Authors:** Jeffrey T. White, Kunj R. Sheth, Aylin N. Bilgutay, David R. Roth, Paul F. Austin, Edmond T. Gonzales, Nicolette K. Janzen, Duong D. Tu, Angela G. Mittal, Chester J. Koh, Sheila L. Ryan, Carolina Jorgez, Abhishek Seth

**Affiliations:** ^1^Scott Department of Urology, Baylor College of Medicine, Houston, TX, United States; ^2^Division of Urology, Department of Surgery, Texas Children's Hospital, Houston, TX, United States; ^3^Department of Urology, Emory University School of Medicine, Atlanta, GA, United States; ^4^Memorial Hermann Health System, Houston, TX, United States

**Keywords:** prune belly syndrome, triad syndrome, Eagle-Barrett syndrome, prenatal intervention, mortality, renal failure, pulmonary hypoplasia, orchiopexy

An author name was incorrectly spelled as Aylin E. Bilgutay. The correct spelling is Aylin N. Bilgutay. The authors apologize for this error and state that this does not change the scientific conclusions of the article in any way.

The original article has been updated.

## Conflict of interest statement

The authors declare that the research was conducted in the absence of any commercial or financial relationships that could be construed as a potential conflict of interest.

